# High frequency CD8+PD-1+ TCR clonotypes isolated from fresh melanomas display anti-tumor activity

**DOI:** 10.1186/2051-1426-3-S2-P42

**Published:** 2015-11-04

**Authors:** Pasetto Anna, Alena Gros, Paul F Robbins, Todd D Prickett, Drew C Deniger, Rodrigo Matus-Nicodemus, Maria R Parkhurst, Jessica S Crystal, Kasia Trebska-Mcgowan, Steven A Rosenberg

**Affiliations:** 1 Surgery Branch/National Cancer Institute / National Institutes of Health, Bethesda, MD, USA; 2 NCI/NIH, Bethesda, MD, USA; 3 NIH, Bethesda, MD, USA; 4 NCI/NIH, Rutgers Robert Wood Johnson Medical School, New Brunswick, NJ, USA

## Background

Adoptive transfer of T cell receptor (TCR) redirected T cells has traditionally targeted shared tumor-specific antigens restricted to common major histocompatibility complexes with the rationale of treating a large population of patients. Recently, tumor infiltrating lymphocytes (TIL) specific for mutated tumor neo-antigens have been shown to play a key role in the clinical response to immunotherapy, suggesting a need for a more personalized therapy. We have shown that anti-tumor reactivity in melanoma principally resides within the CD8+PD-1+ TIL repertoire. Moreover, T cell clones ranking highly in this subset were demonstrated to be tumor-reactive and mutation-specific. Therefore, we hypothesized that the augmented frequency of a T cell clonotype within the CD8+PD-1+TIL could be an indication of tumor antigen-driven clonal expansion, and thus could guide the identification of tumor-reactive TCRs.

## Methods

TCRB sequencing was used to identify the most dominant clonotypes in the CD8+PD-1+ sorted TIL populations. TCRA and TCRB pairs were identified by Adaptive Pairseq of unsorted melanoma and single cell PCR analysis of sorted populations. Full length TCR pairs were reconstructed based on IMGT database [http://www.imgt.org], synthetized and cloned into expression vectors. Open repertoire T cells were transduced with these TCRs and were co-cultured with autologous tumor cell lines and antigen presenting cells expressing tandem minigenes encoding mutated tumor neo-antigens and/or pulsed with corresponding mutated peptides. Tumor and mutation reactivity was determined by IFN-γ ELISA and 4-1BB up-regulation by flow cytometry.

## Results

In 12 melanomas we were able to identify a median value of 217 (range 11-883) TCRA-TCRB pairs by Adaptive Pairseq and 29 (range 9-43) pairs by single cell PCR. For each tumor we reconstructed 7 (range 4-8) TCRs that were ranking within the top 10 clonotypes of the CD8+PD-1+ TIL. Specificity analysis of these reconstructed pairs revealed that in each tumor at least 1 and up to 7 TCRs were tumor reactive.

## Conclusions

Our novel approach allows a rapid identification of TCRs with potential anti-tumor activity. Our data in fact show that high frequency T cell clonotypes within the CD8+PD-1+ population of melanoma TIL are commonly tumor-reactive, suggesting that the frequency of TCRB clonotypes in CD8+PD-1+ TIL may be used to guide identification of tumor-reactive TCRs.

